# Salt Stress-Induced Structural Changes Are Mitigated in Transgenic Tomato Plants Over-Expressing Superoxide Dismutase

**DOI:** 10.3390/biology9090297

**Published:** 2020-09-18

**Authors:** Liliya R. Bogoutdinova, Elena M. Lazareva, Inna A. Chaban, Neonila V. Kononenko, Tatyana Dilovarova, Marat R. Khaliluev, Ludmila V. Kurenina, Alexander A. Gulevich, Elena A. Smirnova, Ekaterina N. Baranova

**Affiliations:** 1 Plant Cell Biology Laboratory, All-Russia Research Institute of Agricultural Biotechnology, Timiryazevskaya 42, 127550 Moscow, Russia; bogoutdinova_lr@rambler.ru (L.R.B.); lazareva-e@yandex.ru (E.M.L.); inna_chaban@rambler.ru (I.A.C.); nilava@mail.ru (N.V.K.); dilovarova@yandex.ru (T.D.); kinggobi@yandex.ru (E.A.S.); 2 Biology Faculty, Lomonosov Moscow State University, Leninskie Gory 1, Building 40, 119991 Moscow, Russia; 3 Plant Cell Engineering Laboratory, All-Russia Research Institute of Agricultural Biotechnology, Timiryazevskaya 42, 127550 Moscow, Russia; marat131084@rambler.ru (M.R.K.); ludmila.kur2208@gmail.com (L.V.K.); 4 Agronomy and Biotechnology Faculty, Moscow Timiryazev Agricultural Academy, Russian State Agrarian University, Timiryazevskaya 49, 127550 Moscow, Russia; 5 N.V. Tsitsin Main Botanical Garden of Russian Academy of Sciences, 127276 Moscow, Russia

**Keywords:** root cap, columella, cell structure, ROS, cytoskeleton, α-tubulin microtubule, salt stress, *Solanum lycopersicum*

## Abstract

**Simple Summary:**

We analyzed the morphological changes in root tip cells caused by the application of iso-osmotic NaCl and Na_2_SO_4_ solutions to tomato plants harboring an introduced superoxide dismutase gene. To study the roots of tomato plants cultivar Belyi Naliv and FeSOD-transgenic line, we examined the distribution of reactive oxygen species and immunodetection of α-tubulin. The differences in the microtubules cortical network between wild type and transgenic plants without salinity were detected. The differences were found in the cortical network of microtubules between control and transgenic plants in the absence of salt stress. While an ordered microtubule network was revealed in the root cells of wild type tomato, no such degree of ordering was detected in transgenic line cells. The signs of microtubule disorganization in root cells of wild type plants were manifested under the NaCl and Na_2_SO_4_ treatment. On the contrary, the cytoskeleton structural organization in the transgenic line cells was more ordered. In addition, the formation of atypical tubulin polymers was observed in response to salt stress. Changes in cell size, due to both vacuolization and impaired cell expansion in columella zone and cap initials, were responsible for the root tip tissue modification.

**Abstract:**

Various abiotic stresses cause the appearance of reactive oxygen species (ROS) in plant cells, which seriously damage the cellular structures. The engineering of transgenic plants with higher production of ROS-scavenging enzyme in plant cells could protect the integrity of such a fine intracellular structure as the cytoskeleton and each cellular compartment. We analyzed the morphological changes in root tip cells caused by the application of iso-osmotic NaCl and Na_2_SO_4_ solutions to tomato plants harboring an introduced superoxide dismutase gene. To study the roots of tomato plants cultivar Belyi Naliv (WT) and FeSOD-transgenic line, we examined the distribution of ROS and enzyme-linked immunosorbent detection of α-tubulin. In addition, longitudinal sections of the root apexes were compared. Transmission electronic microscopy of atypical cytoskeleton structures was also performed. The differences in the microtubules cortical network between WT and transgenic plants without salt stress were detected. The differences were found in the cortical network of microtubules between WT and transgenic plants in the absence of salt stress. While an ordered microtubule network was revealed in the root cells of WT tomato, no such degree of ordering was detected in transgenic line cells. The signs of microtubule disorganization in root cells of WT plants were manifested under the NaCl treatment. On the contrary, the cytoskeleton structural organization in the transgenic line cells was more ordered. Similar changes, including the cortical microtubules disorganization, possibly associated with the formation of atypical tubulin polymers as a response to salt stress caused by Na_2_SO_4_ treatment, were also observed. Changes in cell size, due to both vacuolization and impaired cell expansion in columella zone and cap initials, were responsible for the root tip tissue modification.

## 1. Introduction

Plant protection from the damaging effects of oxidative stress is an urgent problem of plant physiology and biotechnology. Many abiotic and biotic factors cause a stress response in plants, during which the production level of reactive oxygen species (ROS) in cells increases, causing a state of oxidative stress [1]. The different molecular mechanisms and cellular organelles can be targets for ROS in the cells. For example, a ROS homeostasis violation induces a reorganization of the microtubule cytoskeleton, which leads to impaired mitosis and cytokinesis [2,3]. In addition, this process at first caused the microtubules disassembly, and then the formation of abnormal tubulin polymers (macrotubules and paracrystalline aggregates) [2]. Similar changes in the tubulin cytoskeleton were also demonstrated under the various abiotic stresses [4].

Methods of modern biotechnology have enhanced the plant’s protection from stress by introducing genes encoding different components of the antioxidant defense system. Thus, an increase in resistance to oxidative stress was demonstrated in transgenic plants with overexpression of foreign genes encoding superoxide dismutase, ascorbate peroxidase, and glutathione reductase [5]. However, many aspects remain unexplored, related to the peculiarities of the influence of foreign genes expression on the structural and functional cellular organelles organization from transgenic plants under stress.

The role of ROS as molecular regulators of plant signaling systems under the onset of salt stress action, which, when elevated, causes changes in phospholipids and Ca^2+^ content and is accompanied by the production of abscisic acid, is one of the ways to form a cellular response to a stress factor [6,7].

It has been suggested that the key response of cells to the toxic effects of salts is to maintain ionic homeostasis by removing toxic ions [8]. Various salinized areas in the world are characterized by high concentrations of salts, including chlorides, carbonates, and sulfates of magnesium, calcium, potassium and sodium. Sodium and chlorine ions are dominant for saline soils. It was established that the plant’s tolerance to salt stress caused by the action of sodium chloride is controlled by regulating the genes expression of the specific regulatory system SOS (Salt Overly Sensitive). At the same time, significant differences were found in cells belonging to different tissues. Root tissue is the most responsive to salinity [9]. The main components of this activation system are SOS1 (plasma membrane Na^+^/H^+^-antiporter), SOS2 (serine-threonine protein kinase 8), SOS3 (calcium-binding protein p8) [10]. Thus, in *Arabidopsis thaliana* L., an increase in the SOS1 expression [11] with a decrease in SOS3 expression [9] in epidermal cells was shown. However, in the cortex and endoderm cells and endoderm, a high expression level of genes encoding for the calcium-binding protein was revealed only in roots [10]. For the *sos*1 and *sos*2 *Arabidopsis* mutants, it was shown that impaired expression and protein synthesis of the SOS system can cause disorders of the cell cytoskeleton and, consequently, root structure [12]. It is assumed that, since Na^+^ can be the main effector of cortical microtubules depolymerization, the inclusion of the SOS protective system, which is responsible for the removal of Na^+^ ions, leads to the restoration of the cell cytoskeleton structure, although in an altered form, nonetheless capable of supporting cell division and expansion. It is well known that during salinization, osmotic stress, which uses its specific signaling and response mechanisms, has the same damaging effect as the toxic factor conditioned by harmful ions [13]. It was shown that the osmotic pressure of the solution at a concentration of 100 mM mannitol in the culture medium did not affect the organization of the cortical microtubules in mutant plants [14], but led to microtubule depolymerization in control plants, thus providing increased resistance to salt damage. A change in the normal arrangement of interphase microtubules during salt stress was observed in the cells of maize [15,16], alfalfa [4], and tomato [17].

The modification of the cytoskeleton caused by salinity is accompanied by thinning of the network and thickening of microtubule bundles, which is shown for both osmotic and salt effects [3,17]. The bundle’s fragmentation is characteristic under the toxic effect of ions, detected under high osmotic pressure only at strongly inhibitory growth concentrations [4]. Additionally, in some works, the position that we claimed earlier was confirmed, that the cytoskeleton is a highly sensitive target and a marker of damage both under the osmotic and ionic damaging effects [18,19]. Such violations can be either reversible or lead to significant damage, for example, during the formation of crystalline tubulin structures (paracrystals) [3,20], which are likely to form when the interaction of microtubule proteins with regulatory proteins associated with cytoskeletal rearrangement and microtubule relative position is disturbed [21].

Plants use three types of superoxide dismutase (FeSOD, MnSOD and Cu/ZnSOD) in the primary protection of cells from ROS damage. The main function of these enzymes is the enzymatic conversion of such a highly toxic molecule for cells as superoxide into hydrogen peroxide (H_2_O_2_). In turn, peroxide is neutralized by other intracellular enzymes (ascorbate peroxidase, catalase, etc.) to such a harmless compound as water. Chloroplasts are at greatest risk of oxygen toxicity because the molecular oxygen present in photosystem I gains an extra electron and is converted to superoxide by photoreduction. Superoxide dismutase within chloroplasts plays an important role in the prevention of photosynthetic disturbances caused by oxidative damage [22]. The function of this enzyme is duplicated many times in the plant cell. Thus, the *Arabidopsis thaliana* genome contains seven SOD genes (one MnSOD gene, three Cu/ZnSOD genes, and three FeSOD genes), differing in location of expression product [23]. The intracellular localization of FeSOD2 and FeSOD3 was found to be the chloroplast compartment. The localization of FeSOD1, revealed by translational fusion the gene with the GFP gene, indicates its presence in the cytoplasm and nuclear compartments. Antisense expression of FeSOD1 was also found not to result in ultrastructural damage to plastids, in contrast to similar experiments with FeSOD2 and FeSOD3 [24].

Earlier, we obtained a number of transgenic lines with high expression of the target gene FeSOD1, which, in turn, was accompanied by an increase in the enzymatic activity of superoxide dismutase and ascorbate peroxidase [25]. One of these lines, line 19, was selected for this study.

In our work, we analyzed the structural state of the cells and tissues of the root apex, tubulin cytoskeleton in cells of the roots of the tomato original cv Belyi Naliv and its transgenic line 19 expressing the *FeSOD* gene from *Arabidopsis thaliana*, under the action of sodium chloride and sulfate using the methods of light, fluorescence, and transmission electron microscopy (TEM).

## 2. Materials and Methods

### 2.1. Plant Material

The in vitro cultivated tomato (*Solanum lycopersicum* L.) plants of the control cultivar Belyi Naliv (Wild Type) and transgenic line 19 expressing the superoxide dismutase *FeSOD* gene from *Arabidopsis thaliana* (L.) Heynh. [26] were the object of comparative study. Cloned tomato plants were grown in a growth chamber for 14 days at 25 ± 2 °C under 16/8 h light/dark diurnal cycle with a light intensity of 160 μmol photons m^−2^ s^−1^, and air humidity of 60–70%. Then, the plants were placed on ½ MC medium with the addition of salt solutions (99 mM NaCl and 87 mM Na_2_SO_4_) in iso-osmotic (‒0.4 MPa) concentration, and cultivated for 7 days under the same temperature/light/humidity conditions. The concentration was chosen based on the results of preliminary experiments as caused by a strong delay in seedling growth [27]. After that, pieces of plant leaf were fixed for various microscopic analyses.

### 2.2. Intravital Fluorescence Microscopy

The tomato root tip (4–5 mm) of all in vitro cultivated samples of control and FeSOD-trangenic line 19 7-days-after were separated and placed on a glass slide in a drop of water. We used an aqueous solution of Carboxy-H2DFFDA (Thermo Fisher Scientific, Waltham, MA, USA) for intravital ROS visualization in cells, at a concentration of 25–50 nM after 30 min incubation time. The stained pieces were washed three times with sterile water.

Intravital root preparations were analyzed using an Olympus BX51 fluorescence microscope (Olympus Corporation, Tokyo, Japan), with magnification ×10, at a wavelength of 490 nm. Images were obtained using a Color View II digital camera (Soft Imaging System, Munster, Germany).

### 2.3. Immunocytochemistry of Tubulin Cytoskeleton in Root Cells

To analyze the structural organization of the tubulin cytoskeleton in root cells, the tomato plants root tips of control and transgenic line grown in conditions without salt addition and under the action of salts were used.

### 2.4. Fixation and Preparation of Macerated Root Cells

Root tips (4 mm) after 7 days cultivation in salinity imitating conditions were cut off for fixation. The detached fragments were fixed in a freshly prepared solution of para-formaldehyde (Sigma-Aldrich, St. Louis, MO, USA) on a PHEM buffer (pH 6.9; including 60 mM PIPES, 25 mM HEPES, 10 mM EDTA and 2 mM MgCl2 (all components by Sigma Aldrich, St. Louis, MO, USA) for 2 h at room temperature. The fixator was removed by 2× washing in a PHEM buffer. For maceration, the root pieces were treated with a solution of 2% cellulase in a Na-acetate buffer (pH 5.0), and then the samples were transferred to a PHEM buffer. Next, the roots were transferred on a coverslip in a buffer drop and split to separate cells with metal needles. Then the samples were dried for 24 h in a refrigerator at 4 °C.

### 2.5. Tubulin Immunolocalization

Preparations were washed with PHEM-buffer. Next, a drop of PHEM-buffer containing 0.5% Triton X-100 and 5% DMSO was applied to the sample, and then a preparation was incubated in 5% goat serum for 30 min to block non-specific binding to antibodies. The first antibodies used were murine monoclonal antibodies to tubulin DM1α (Sigma Aldrich, St. Louis, MO, USA) at a dilution of 1: 200 in 10 mM Tris buffer (pH 7.6) with the addition of 0.1% BSA for 16 h at 20 °C. After washing twice in 10 mM Tris buffer (pH 7.6) and 20 mM Tris buffer (pH 8.2), the preparations were incubated with second goat antibodies to mouse IgG conjugated with Alexa-488 (Thermo Fisher Scientific, Waltham, MA, USA) in dilution 1:400 for 45 min at 37 °C. To reveal the nuclei, the prepared preparations were stained with DAPI (Sigma Aldrich, St. Louis, MI, USA), then mounted in Moviol 4–88 (Hoechst, Frankfurt am. Main, Germany). The preparations were placed in a 4 °C refrigerator and assessed under an Axiovert 200M microscope (Zeiss, Wetzla, Germany) with a Neofluar 100/1.24x objective under epifluorescence illumination with a set of filters (excitation 450–480 nm and emission 515–565 nm for Alexa Fluor; and with excitation 365 nm and a peak emission of 420 nm for DAPI). For immunocytochemical and electron microscopic studies, meristematic cells from the central zone of root apex belonged to the interphase (G1-S) of the cell cycle were used. Images of each cell were obtained in several optical sections (at the level of the nucleus and the level of the edge of the cell) using an AxioCam HRm digital camera and processed in Adobe Photoshop 7.0.

### 2.6. Transmission Electron Microscopy

For ultrastructural analysis, plant root tips up to 3 mm long were separated. Samples were fixed in a 2.5% glutaraldehyde solution in 0.1 M Sorensen phosphate buffer (pH 7.2) with the addition of 1.5% sucrose. After washing from the fixing mixture, the samples were post-fixed with a 1.0% solution of osmium tetroxide (OsO4), dehydrated in ethanol with increasing concentration (30, 50, 70, 96, and 100%), propylene oxide and encapsulated with Epon–Araldit epoxy resins. Semi-thin and ultra-thin sections were prepared using the LKB–III ultramicrotome. Sections were contrasted with a 1% aqueous solution of uranyl acetate and lead citrate accordingly Reinolds, and analyzed at ×15,000 magnification using an H-500 transmission electron microscope (Hitachi, Tokyo, Japan). Images were processed in Adobe Photoshop 7.0.

### 2.7. Statistical Analysis

Statistical analysis of the dates was performed using Fisher’s and Duncan’s tests with the program AGROS (version 2.11). To estimate the average number of columella cell layers, we used 50 transverse sections of root tips from 5 independent plants for each variants.

## 3. Results

**Figure 1:**
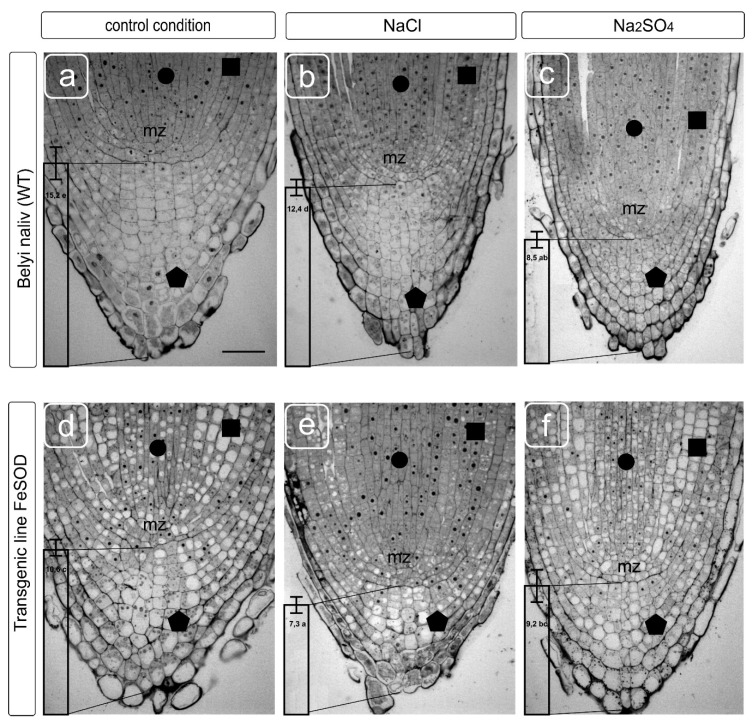
Transverse sections of root tips from wild-type (WT) (**a**–**c**) and transgenic tomato (**d**–**f**) plants grown without and supplemented with NaCl and Na_2_SO_4_. Modification of meristem and cap cells while the imitation of salinity effects in vitro culture. Responsiveness of the following parameters to salt stress is shown: the size and shape of the columella zone cells from the root cap (indicated by a vertical column with an average number of columella cell layers) and the initials extending from the meristematic zone (mz) from the root tip. Symbols: circle–stele cells, square–cortex cells, pentagon–columella cells. Scale bar: 50 μm. Transverse sections of root tips show the average number of columella cell layers. Values followed by the same letter significantly do not differ by Duncan’s test (α = 0.05) (50 transverse sections of root tips from five independent plants).

The root tips of the tomato plants cv Belyi Naliv (WT) and the transgenic line 19 obtained on this variety basis corresponded to the type of structural organization typical for tomatoes (Figure 1). They have a clearly identifiable cap with columella cells. There were also lateral cells of the cap and epiblema cells differing in size, location and internal structure from columella cells, and covering the surface of the young root tip to the layer of exfoliation. Normally, the root cap has a characteristic structure and contains from 14 to 16 layers of columella cells from the meristem zone to the apical point. Layers close to the surface have signs of programmed cell death with characteristic densification of the cytoplasm, the disappearance of vacuoles and pronounced plasmolysis [28]. The root meristem has a clear-cut structure that makes it easy to identify the cells of the developing tissues of the exoderm, cortex, pericycle and stele (central cylinder) with a common for normal conditions, reduced level of vacuolization in small cells with large nuclei and nucleoli located in the center of the cells (Figure 1). NaCl caused a change in the morphology of the root apex, a decrease in the layers of columella and cortex cells and cell sizes. The meristem cells had a denser content, the nucleoli in cell nuclei were enlarged, and a noticeable change in the vacuolization of the lateral cells of the cap, columella and the initials of the cellular tissues adjacent to the meristem was observed. Na_2_SO_4_ caused a significant increase in cell density both in the cap and in the meristematic zone and root initials.

**Figure 2:**
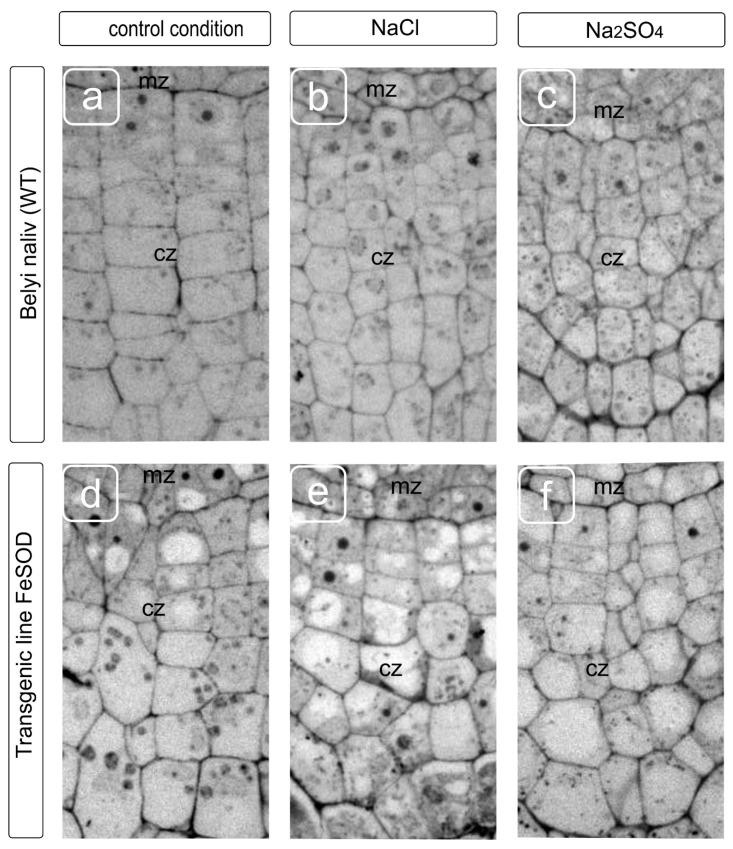
Fragments of transverse sections of root tips showing the zone of cap initials forming apex columella from WT (**a**–**c**) and transgenic tomato (**d**–**f**) plants grown without (**a**,**d**), and supplemented with the NaCl (**b**,**e**) and Na_2_SO_4_ (**c**,**f**). Modification of the cell walls of root apical meristem and cap cells of the tomato root tip is shown. The thickness and location of adjacent layers related to the cap initials, the transformation of the size and shape are visible as the most sensitive targets for salts treatments. Symbols: mz–meristem zone; cz–columella zone.

A statolith formation and starch deposition in them is an important indicator of the cap development and, partially, of the outer cortical cells development, represented by epidermal and sub-epidermal tissue. The amount of starch depends on sucrose from the aboveground part due to phloem transport, which is converted into starch deposits of statoliths. The statoliths provide gravitropic reactions (vertical growth and bending), which coincide with the auxins modification and sugars transport [29]. A change in the number of cell layers and their relative arrangement supports the assumption about a decrease in the intake of starch in columella cells by salt exposure, as shown earlier [30]. The change in shape and a decrease in the size of cap cells in WT caused by NaCl and Na_2_SO_4_ treatments were accompanied by a redistribution of the cytoplasmic compartment, a more chaotic arrangement of vacuoles and a decrease in their size under application of both salts. It seemed that Na_2_SO_4_ had been causing some cytoplasm compaction and cell wall thickening (Figure 2). FeSOD-transgenic plants were characterized by a perturbation of the cell arrangement, and an increase in the variety of sizes in a cell row, in which there is no gradual increase in cells towards the distal zone of the root cap (Figure 2d). The cell row ordering was retained with some disturbance in the shape of the cells under the NaCl and Na_2_SO_4_ treatments. There were no differences in cell size, whether salt treatment was used or not (Figure 2d–f). In our opinion, some thickening of the cell walls was observed in WT under the Na_2_SO_4_ action, and in transgenic line 19 both under the action of salts and in the absence of a stress factor.

Cells in the zone of the cap’s initials, adjacent to the root meristematic zone, were characterized by the central location of the nucleus and had several small vacuoles beginning to form under without stressful conditions. The cells had a regular rectangular shape. They are arranged in rows one above the other, and the farther the cell is from the initials, the more it increases in width and, it seems, contains only one central vacuole in the fourth layer from the meristem. The nucleus and cytoplasmic organelles are shifted to the periphery of the cells and are located along the cell walls, while in older layers, large columella cells contain starch-filled statoliths and the nucleus in the upper part of cells. Presumably, NaCl and Na_2_SO_4_ caused the earlier vacuolization of cells, and if NaCl did not prevent the fusion of vacuoles into one large vacuole, then the smaller vacuoles were observed under the Na_2_SO4, the uniform distribution of organelles in old (distal from the meristem) cells was preserved, and the formation of a central vacuole was blocked. The features of the fusion of vacuoles are probably of separate interest. The acceleration of the vacuole fusion process could be observed in columella cells in plants of the FeSOD-transgenic line cultivated without salinity. In this case, the displacement of the organelles and nucleus to the cell periphery was observed already in the second or third layer situated distally from the initials. Cell arrangement did not have the proper degree of ordering of the gradual location from the initials to the periphery, which is probably caused by the consequences of a disturbance in division and expansion processes. An orderliness of the layers and a gradual increase in cell size were maintained under NaCl and Na_2_SO_4_ treatments. However, the location of organelles, nuclei, and vacuoles in the cells differed significantly from layer to layer, and from row to row. Apparently, this can be explained by a violation of the membrane fusion processes, division of compartments, and, probably, restrictions on the movement of the cytoplasm. Such perturbations associated with the dynamic transformation of the cytoskeleton are well studied and are related to impaired osmotic homeostasis [20,31].

**Figure 3:**
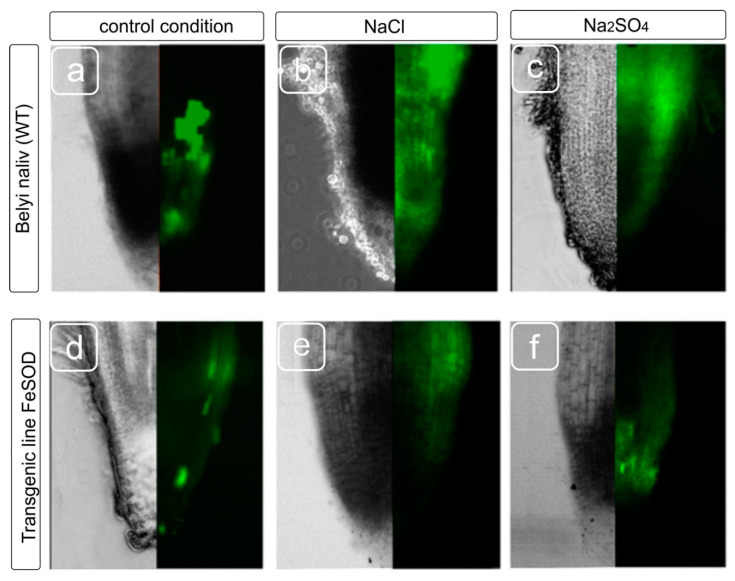
Distribution of reactive oxygen species (ROS) in the different zones of tomato roots. WT (**a**–**c**) and transgenic line 19 (**d**–**f**) plants were grown without, and with the addition of NaCl and Na_2_SO_4_. A change in the localization of ROS in the meristem and cap cells of the root tip is shown as a modification of fluorescence.

Staining the roots of tomato plants with a fluorescent dye on ROS production showed that the ROS were detected in all root tissues upon salinity stress, however, the staining intensity varied in cells from different zones. Since not all root zones were equally stained for ROS, we estimated the distribution of cells with an increased level of ROS production in different zones of the roots (Figure 3). Root zones with bright fluorescence, a moderate level of fluorescence, a low level and the absence of luminescence were noted. It turned out that both the original cultivar and the transgenic line showed intense fluorescence under both salt treatments. Fluorescence was observed in the division and elongation zones, moreover, the fluorescence in the root of the transgenic line was moderate intensity, and the fluorescence of WT was brighter. At the same time, in plants exposed to salts, an increase in the ROS level is most noticeable in epidermal and cortex cells, and to a lesser extent, in the stele region compared to WT plants that did not experience salt stress. Transgenic line 19 demonstrated weaker fluorescence compared to the WT (Figure 3). Thus, to study the effect of oxidative stress induced by salinity, epidermal and cortical cells from both the division and elongation zones are more preferred for studying ROS induction caused by stressful effects than the cells from the root cap.

The cytoskeleton is one of the targets for ROS [32,33]. The experimental violation of ROS homeostasis in the plant cells causes the microtubules disassembly, and then the formation of abnormal tubulin structures (macrotubule and paracrystalline aggregates) [6]. However, it remains unclear whether similar changes in the tubulin cytoskeleton occur under the abiotic stressful effects, accompanied by activation of ROS production. We examined the state of the microtubule system under the NaCl and Na_2_SO_4_ stress in the root tip cells from WT and FeSOD-transgenic line 19 tomato plants using immunocytochemistry and TEM.

### 3.1. Control cv Belyi Naliv (WT)

**Figure 4:**
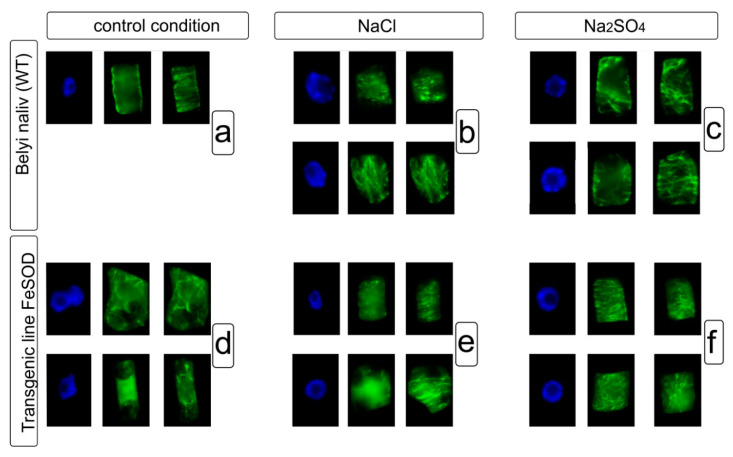
Tubuline cytoskeleton in cell cycle interphase of root cells from WT (**a**–**c**) and transgenic tomato (**d**–**f**) plants grown without, and with the addition of NaCl and Na_2_SO_4_. Nuclei were stained with DAPI (**blue**); microtubules were detected using antibodies to α-tubuline (**green**). While the bundles of microtubules are located in the cortical cytoplasm, microtubules are not visible in the perinuclear region in the interphase cells. Tomato root cells have obvious multiple lesions in the location of microtubules under the NaCl, Na_2_SO_4_. Cells form multiple chaotically arranged bundles and do not maintain peripheral position, which indicates violations in the process of cytoskeleton transformation, as a highly dynamic structure.

Microtubules form a cortical network consisting of bundles that are parallel to each other and oriented perpendicular to the cell growth axis (Figure 4a) in conditions without salt stress. The application of NaCl resulted in cortical microtubules consisting of bundles that were parallel to each other and oriented perpendicular to the cell growth axis in root tip cells (Figure 4b). The cortical microtubules had signs of disorganization, such as a disturbance of the parallel bundle organization, the formation of microtubule convergence centers, and the fragmentation, shortening and thickening of microtubule bundles (Figure 4b) under NaCl. Similarly, the microtubule network chaotization and formation of converging centers, asa well as the change in the orientation of microtubule bundles with respect to the growth axis are clearly manifested in cells with the Na_2_SO_4_ treatment (Figure 4c).

### 3.2. FeSOD-Transgenic Line 19

Root cells from transgenic tomato line 19 had an atypical morphology even without salt stress. These cells showed the vacuolization of cytoplasm that led to the displacement and deformation of the nucleus (Figure 4d). In addition, the organization of the microtubule system is significantly impaired in these cells. Microtubules are short, rare, and chaotically oriented, and an ordered cortical network is not pronounced.

The structural organization of the microtubule network in the root cells from the transgenic line was more ordered under NaCl stress than under non-saline conditions. This was manifested in the fact that microtubules were more numerous and formed the ordered or chaotic networks in the cortical cytoplasm. Along with this, there were cells with rare, short and thick bundles in the cortical cytoplasm (Figure 4e).

A cortical network with bundles of microtubules arranged parallel to each other was detected in cells exposed to Na_2_SO_4_. Cells with bundles heterogeneous in length and density, and forming the converging were also observed. In addition, along with ordered bundles, the cells contained short and/or chaotically oriented microtubules (Figure 4f).

Thus, abiotic stress (salinity) causes certain rearrangements of microtubules (tubulin cytoskeleton) of plants: (1) disturbance of the ordered organization of the cortical network microtubule such as chaotization, change in orientation relative to the growth axis; possibly, this is the reason for the formation of microtubule converging centers, although it cannot be ruled out that such centers indicate the initiation of the growth for new microtubules; (2) network becomes less dense, possibly due to the depolymerization of the microtubule sub-population; (3) formation of thick and dense bundles; (4) appearance of fragmented and shortened microtubules bundles. When comparing the response to the salinity of the microtubule system in the plant roots of WT and FeSOD-transgenic line tomato plants, it was found that this response of microtubules in the root cells of WT tomato to such an abiotic factor as salinity was manifested in different variants of disorganization. Transgenic line 19 initially had an abnormal microtubule system, but upon salt exposure, partial phenotypic “recovery” occurred that was manifested in an increased number of bundles and the formation of cortical networks (both ordered and chaotic). A number of studies have suggested that when the ROS homeostasis in cells changes, the tubulin cytoskeleton passes into another structural state by assembling atypical tubulin structures [34]. However, to date, there is no evidence that similar changes in the tubulin cytoskeleton occur under any abiotic stress.

Using TEM, we also analyzed the state of the tubulin cytoskeleton in the root cells of the control (WT) and transgenic tomato plants under NaCl and Na_2_SO_4_ treatments.

**Figure 5:**
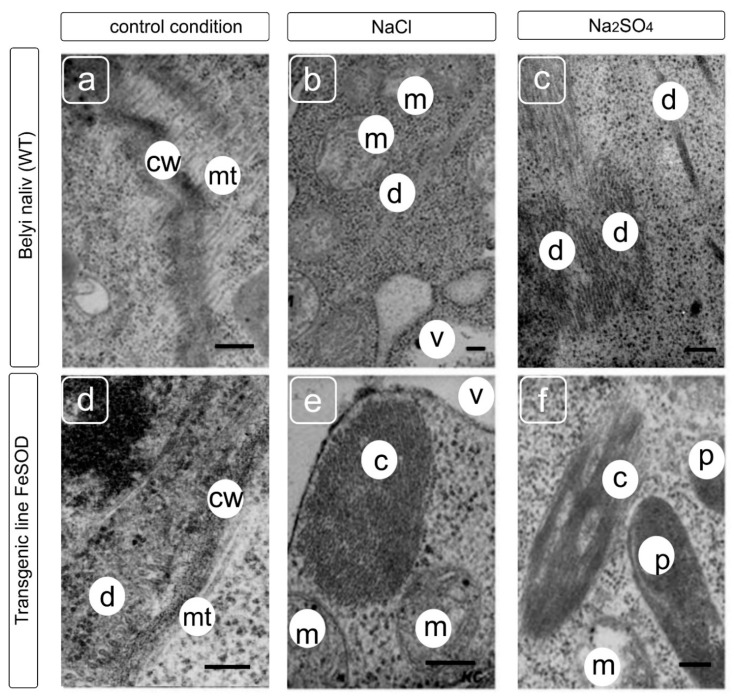
Ultrastructure of root cell from WT (**a**–**c**) and Fe-SOD-transgenic tomato (**d**–**f**) plants grown without and supplemented with NaCl and Na_2_SO_4_. Symbols: mt—microtubules, cw—cell wall, m–mitochondrion, v—vacuole, p—plastid, c—atypical cytoskeleton structure. Scale bars—250 nM.

We did not find abnormal formations of the cytoskeleton elements in the cell cytoplasm of WT tomato grown without salt exposure (Figure 5a). Only single or bundled microtubules, generally located near the cell wall, were observed in the cells. In addition, upon salinity, in the root cells of WT, along with microtubules located at a somewhat greater distance from the plasma membrane (compared to conditions without salt exposure), under the NaCl salinity, more densely packed tubular bundles/strands were revealed. We observed structures similar to atypical tubulin polymers under the Na_2_SO_4_ (Figure 5f) [34,35].

Microtubules were detected in the cortical cytoplasm and were arranged parallel or perpendicular to the cell wall in root cells of FeSOD-transgenic line 19 during salt exposure (Figure 5d–f). However, in the same cells, in addition to microtubules, atypical elements of the cytoskeleton were present, which were absent in plants that were not exposed to salt stress. Fibrillar–tubular strands were revealed in root cells of transgenic tomatoes under the NaCl stress (Figure 5e). Bundles consisting of clusters of tubular and orderly packed structures were present in the cells under Na_2_SO_4_ (Figure 5f). Unlike microtubules, these tubular structures did not have a central lumen, or it was significantly smaller in diameter. It can be seen on the cross-sections that the tubular structures in the bundle have an ordered cellular packing characteristic of tubulin paracrystals [12,31,36].

## 4. Discussion

A tolerance to stressful conditions is known to correlate with the activity of enzymes involved in ROS detoxification [37]. The application of plant genetic engineering makes it possible to introduce heterogenous genes into plants, which make it possible to quite successfully withstand the damaging consequences of abiotic stresses that are accompanied by an increase in the ROS level. Thus, the gene that encodes chloroplast-localized Cu/Zn superoxide dismutase was protected from oxidative stress caused by exposure to high light intensity [38]. Generally detrimental to metabolism, superoxide and hydrogen peroxide may serve useful functions if rigorously controlled and compartmentalised [39]. The Sod1(from mangrove plant *Avicennia marin*) transgenic plants were more tolerant to methyl viologen mediated oxidative stress and withstood salinity stress of 150 mM NaCl for a period of eight days while the untransformed control plants wilted at the end of the stress treatment in hydroponics [40]. In another study, the increases in SOD activities as low as 0.15-fold could also significantly enhance salt tolerance in transgenic poplar plants with the MnSOD gene (TaMnSOD) from *Tamarix androssowii*, suggesting an important role of increased SOD activity in salt tolerance [41]. Additionally, *A. thaliana* transgenics overexpressing cytosolic CuZn-superoxide dismutase (PaSOD) from *Potentilla atrosanguinea*, cytosolic ascorbate peroxidase (PaSOD) from *Rheum australe* and dual transgenics overexpressing both the genes were developed and analyzed under salt stress. In comparison to wild-type (WT) or single transgenics, the performance of dual transgenics under salt stress was better with higher biomass accumulation and cellulose content at 100 mM salt stress [42]. However, in numerous studies with transgenic plants, no attention has been paid to structural conversions manifesting at the cellular level.

The cap is a sensitive model that responds quickly to the action of most edaphic factors. It provides an orientation of root growth, location of roots in space and a number of other functions. These functions are provided due to a complex mechanism associated with the transport of sugars from the aboveground part, the redistribution of starch in the tissues and root zones, and their hormonal regulation. This is reflected in the structural organization of the meristematic zone, columella, and lateral cells of the root cap [43].

In the present study, the negative effect of salts led to a marked inhibition of both the columella cell formation and the injury of the cell shape and vacuolization (Figure 1). This effect is characteristic of adverse factors, such as drought, high or low (not typical for plants) pH values, and the presence of toxic metal ions. Sensitive systems of cell growth and division lead to changes in fine regulation processes, primarily affecting the location of cellulose microfibrils and other elements of the cell wall, and disruption of cell expansion and, in total, growth directions [44]. This had been reflecting in the violation of cell shape under sodium chloride and sulfate treatments. These processes are directly related to the transformations of the tubulin cytoskeleton. The disruption of the preprophase band location leads to the displacement and sometimes deformation of the normal phragmoplast location, leading to the disruption of the cell division direction, subsequent growth [45], and interaction between cells and cell transport. When the mechanism of tubulin microtubule redistribution—located along the cell wall—is damaged, uneven distribution occurs, along with the formation of seals and a curving of the cell walls, and cells of irregular shape are formed [46]. Additionally, cells appear that have significantly different sizes and, accordingly, are unable to maintain characteristic transport, programmed cell death, and exfoliation necessary to ensure growth, surface lubrication and root movement in the substrate. An injury of the cytoskeleton leading to a disturbance in the shape of cells (Figure 1 and Figure 2) accompanies a change in the morphology of the meristematic cells in the present study (Figure 4 and Figure 5). In some cases, these processes are accompanied and provided by autophagy [47].

The vacuolar compartment contributes to the adaptive mechanisms of structural cell transformations and tissues during salinity. The most important responses of the vacuolar system to harmful influences can be: disruption of fusion, when the resulting vacuoles remain in the places of their formation; displacement of the vacuole relative to the characteristic central location; shape change from rounded to irregular. All these external manifestations can be the result of various violations of osmotic homeostasis, as well as a violation of the cytoplasm ability to move due to a decrease in fluidity or the appearance of obstacles in the form of the cytoskeleton abnormal transformations. In this work, the activation of the vacuolization processes in meristem and cap cells during ectopic overexpression of Fe-dependent superoxide dismutase was revealed, which indicates an important role of ROS in the regulation of the vacuole initiation processes and the regulation of their fusion and growth. Changes in the cell vacuolization of the root tip and meristem were detected under salts both in control tomato plants and in FeSOD-transgenic line 19 (Figure 1 and Figure 2). It was shown that sodium chloride could significantly reduce this effect in the stele tissues, while the isosmotic concentration of sodium sulfate reduced the severity of this process, but this did not have a tissue-specific effect.

An increase in the ROS pool was the expected effect of salt exposure (Figure 3). We expected a decrease in the ROS level in the root tissues of the FeSOD-transgenic line that was confirmed both for normal conditions without salt exposure and for salt treatment. The amount of ROS increases when plants are exposed to cold, salinity, drought, floods, and herbicide treatments [47], and, in general, during almost every type of biotic or abiotic stress [48]. Ion imbalance and hyperosmotic stress caused by the use of NaCl and Na_2_SO_4_ result in further growth inhibition and various damage to macromolecules during the formation of ROS [49]. It can be assumed that the growth of H_2_O_2_, caused by the functioning of Fe-dependent superoxide dismutase, led to an increase in the adaptive cell capabilities of transgenic tomato plants and the preservation of their growth and development despite inhibition by toxic ions.

Cortical microtubules play a vital role in plant cell growth [50], and are also involved in responses to biotic [51] and abiotic stress [15]. They help withstand salt stress by regulating cell growth and binding to cell compartments. The reorganization and re-polymerization of cortical microtubules is the response of plant cells to salt stress. In addition, osmotic stress caused the reorganization of microtubules in corn root cells, their reorientation inhibited cell expansion [15]. Moreover, the effect of ROS on the tubulin cytoskeleton has been shown [2]. In our study, we examined the effect of NaCl and Na_2_SO_4_ on the system of cortical microtubules in the root cells of control (WT) and FeSOD-transgenic tomato plants. Initially, we analyzed the root cells cytoskeleton of non-transgenic and transgenic plants grown without salt exposure. It was found that in this case, the microtubule system in the interphase tomato root cells was different. In cells of WT, a cortical network of microtubules was visible, in which the bundles are located parallel to each other, and perpendicular to the cell growth axis. The cells themselves had a typical shape characteristic of the cells in these layers. A different situation was found in the root cells of the transgenic tomato line 19. Here, the organization of the microtubule system was disrupted. Thus, the study revealed short, rare, chaotically oriented microtubules. In line 19, no ordered cortical network was found in comparison with WT. Another difference is the atypical cell morphology s, in which an increased degree of vacuolization is shown, which, apparently, could become one of the reasons for the displacement and deformation of the nucleus. Further, the network of cortical microtubules in root cells of WT and transgenic tomato plants was compared under NaCl stress. Substantial differences between the elucidated variants were noticed. Thus, the parallel organization of cortical bundles in WT root cells, characteristic of conditions without sodium chloride addition, showed signs of disorganization. Network chaotization, the formation of microtubule converging centers, and the fragmentation, shortening and thickening of the bundles were found. On the contrary, in transgenic tomato line 19, the structural organization of the microtubule network in root cells was more ordered upon the addition of NaCl than under non-saline conditions. This was manifested in the observation that there were more microtubules in the cytoplasm of root cells than in non-saline conditions, and they formed an ordered or chaotic network in the cortical cytoplasm. Along with this, cells with rare, short and thick bundles were sometimes revealed. Similar changes in plant root cells were observed when plants had been growing in the presence of Na_2_SO_4_. In the WT, the microtubule network chaotization of the formation of convergence foci and the change in the microtubule bundle orientations relative to the growth axis are clearly manifested (Figure 4). The cells retain their characteristic morphology. Root cells in transgenic tomato line 19, in contrast to the non-saline conditions, had the correct shape. Nevertheless, a cortical network with bundles of microtubules arranged parallel to each other is revealed in the cells. Bundles of different lengths and densities form converging centers. At the same time, along with ordered bundles, short and/or chaotically oriented microtubules are observed in root cells.

Some researchers have suggested that when ROS homeostasis in cells changes, the tubulin cytoskeleton passes into another structural state by assembling atypical tubulin structures [34]. The assembly of the atypical tubulin polymers is an adaptation to various stresses [52,53]. Atypical tubulin polymers can be considered as more stable structures than macrotubules. They are formed and retained in cells during hyperosmotic stress [52,53], treatment with aluminum and disruption of homeostasis, including due to change in ROS synthesis. The obtained data indicate that salt stress induces in the transgenic root cells the assembly of atypical elements of the cytoskeleton, which are morphologically identical to tubulin aggregates formed during experimentally induced ROS imbalance [2]. If we assume that the ectopic overexpression of the *FeSOD* gene in transgenic tomato plants leads to an increase in the tolerance to stress factors, the formation of atypical tubulin polymers under stress conditions accompanied by an increase in the ROS level is a manifestation of such a protective reaction [47]. It is assumed that the interaction of ROS and microtubules provides the perception of a stressful situation and triggers the corresponding response. Tubulin cytoskeleton reorganization plays an important role during this process. The depolymerization of the microtubule network first occurs in plant cells during salinity, and then new microtubules are collected, forming the stable systems which contribute to an increase in plant salt tolerance [12]. We believe that after abiotic stress, such as salinity inducing microtubule depolymerization (complete or partial) in the root cells of FeSOD-transgenic tomato plants, the atypical tubulin polymers assemble as a characteristic nonspecific response to abiotic stresses, which are characterized by a significant enhancement to oxidative stress by increasing the ROS pool. It is possible that there is a priming effect leading to the formation of a number of responses due to changes in gene expression, transport of sugars and phytohormones. This effect provides nonspecific protection against stress damage [54]. This was confirmed by experiments with a brief application of moderate H_2_O_2_ levels, which significantly enhanced oxidative stress tolerance by elevating the antioxidant status of the plant cells and tissues [55]. The results presented in our study demonstrate that noticeable changes in intracellular structures were observed in the root cells of transgenic tomato owing to the ectopic superoxide dismutase expression, which strongly altered the oxidative status of cells.

## 5. Conclusions

A variation in the oxidative status of a cell due to heterologous expression of Fe-dependent superoxide dismutase leads to an alteration in the structural and functional organization of the root tip, a change in the ROS localization in the cap, cortex and stele, and a disruption in the dynamic transformations of the microtubule cytoskeleton, contributing to a change in cell vacuolization, primarily of the columella and cortex. The introduction of the heterogenous superoxide dismutase gene into the tomato genome made it possible to significantly mitigate the harmful effects of salt stress on intracellular structures caused by the application of sodium chloride and sulfate.

The determination of the functional cell state using intravital markers would be effectively used in future estimations for the rapid assessment of the oxidative status of plant root cells grown under various stress factors.
